# Deciphering the Allosteric Effect of Antagonist Vismodegib on Smoothened Receptor Deactivation Using Metadynamics Simulation

**DOI:** 10.3389/fchem.2019.00406

**Published:** 2019-06-04

**Authors:** Xiaoli An, Qifeng Bai, Fang Bai, Danfeng Shi, Huanxiang Liu, Xiaojun Yao

**Affiliations:** ^1^State Key Laboratory of Applied Organic Chemistry and Department of Chemistry, Lanzhou University, Lanzhou, China; ^2^School of Pharmacy, Lanzhou University, Lanzhou, China; ^3^State Key Laboratory of Quality Research in Chinese Medicine, Macau Institute for Applied Research in Medicine and Health, Macau University of Science and Technology, Macau, China

**Keywords:** smoothened receptor, vismodegib, cholesterol, metadynamics simulation, allosteric inhibition mechanism

## Abstract

The smoothened receptor (Smo) plays a key role in Hedgehog (Hh) signaling pathway and it has been regarded as an efficacious therapeutic target for basal cell carcinoma (BCC) and medulloblastoma (MB). Nevertheless, the resistance mutation and active mutants of Smo have put forward the requirement of finding more effective inhibitors. Herein, we performed metadynamics simulations on Smo bound with vismodegib (Smo-Vismod) and with cholesterol (Smo-CLR), respectively, to explore the inhibition mechanism of vismodegib. The simulation results indicated that vismodegib-induced shifts of TM5, TM6, and TM7, which permitted the extracellular extension of TM6 and extracellular loop3 (ECL3) to enter the extracellular cysteine-rich domain (CRD) groove. Therefore, an open CRD groove that has not been noticed previously was observed in Smo-Vismod complex. As a consequence, the occupied CRD groove prevents the binding of cholesterol. In addition, the HD and ECLs play crucial roles in the interaction of CRD and TMD. These results reveal that TM5, TM6, and TM7 play important roles in allosteric inhibition the activation of Smo and disrupting cholesterol binding by vismodegib binding. Our results are expected to contribute to understanding the allosteric inhibition mechanism of Smo by vismodegib. Moreover, the detailed conformational changes contribute to the development of novel Smo inhibitors against resistance mutation and active mutants of Smo.

## Introduction

The Hedgehog (Hh) signaling pathway plays central roles in the animal development and stem-cell function, linking to cell growth, and differentiation, with normal roles in embryonic pattern formation and adult tissue homeostasis and pathological roles in tumor initiation and growth (Pasca di Magliano and Hebrok, 2003; Lum and Beachy, 2004; Rohatgi and Scott, 2007). The smoothened receptor (Smo) serves as one of the key proteins in this signaling pathway, the phosphorylation, stabilization, and accumulate of Smo cause signal to intracellular effectors (Denef et al., 2000; Kalderon, 2000).

Smo has been regarded as an efficacious therapeutic target for basal cell carcinoma (BCC) and medulloblastoma (MB) (Kim et al., 2013). Nevertheless, therapeutic challenges remain in the tumors that acquire resistance to Smo antagonists (Rudin et al., 2009; Yauch et al., 2009; Dijkgraaf et al., 2011; Sharpe et al., 2015a), also in cases of active mutants of Smo that were reported as oncogenic drivers (Reifenberger et al., 1998; Sweeney et al., 2014). So the emergence of resistant mutations and active mutations has raised the need for the discovery of novel Smo inhibitors or novel target sites of Smo.

As a member of the F family of GPCRs, the structure of Smo, so as frizzled (FZD) receptors (Huang and Klein, 2004), contains an extracellular domain (ECD) composed of an extracellular cysteine-rich domain (CRD) and a linker domain (192-222), a seven-transmembrane helical domain (TMD) and an intracellular carboxy-terminal domain (ICD). The CRD and TMD are connected by linker domain (Schulte, 2010; Byrne et al., 2016). Zhang et al. published the structures of multiple domains of Smo and the linker domain called hinge domain (HD) (191-220) (Zhang et al., 2017). Smo possesses two separable ligand-binding sites, one in the TMD and another in the CRD (Sharpe et al., 2015b). The linker domain and TM helices form the deep hydrophobic TMD binding pocket, targeted by allosteric inhibitors and activators, including LY-2940680 (Bender et al., 2011; Wang et al., 2013), cyclopamine (Chen et al., 2002a; Weierstall et al., 2014), SANT-1 and SAG1.5 (Chen et al., 2002b; Miller-Moslin et al., 2009; Wang et al., 2014), and the anticancer drug vismodegib (Robarge et al., 2009; Byrne et al., 2016). Vismodegib has been used to treat advanced basal cell cancer in clinical practice. Another ligand-binding site is located in the hydrophobic groove of the CRD (Nachtergaele et al., 2013; Nedelcu et al., 2013), targeted by cholesterol (Cooper et al., 2003; Huang et al., 2016), 20(S)-hydroxycholesterol (OHC) and other cholesterol analogs (Dwyer et al., 2007; Nachtergaele et al., 2012), that activate Smo in Hh signaling pathway (Nedelcu et al., 2013). Cholesterol was regarded as the endogenous activator of Smo (Huang et al., 2016).

Previous biochemical studies have shown that sterols binding to CRD groove site activate Hh signaling pathway. Binding of an agonist or antagonist to the TMD-site can activate or inactivate Hh signaling pathway. In addition, the vismodegib binding to the TMD-site results in loss of cholesterol from the CRD–linker domain–TMD interface (Byrne et al., 2016). Zhang et al. have demonstrated that TM6, extracellular loop 3 (ECL3) and the HD play a central role in signal transmission, and their structures reveal a precise arrangement of TMD, HD, and CRD. This structure enables allosteric interactions between the three domains that are important to ligand recognition and receptor activation (Zhang et al., 2017). Yet, the detailed mechanisms of how vismodegib allosterically inhibits the activation of Smo and binding of cholesterol remain unknown.

The crystal structures of Smo binding with vismodegib and cholesterol have similar conformation, both in inactive states. We have little known from the static crystal structures about the conformational variation in Smo upon binding different ligands. The computational methods such as molecular dynamics (MD) simulations can provide the information about the dynamic process of conformational changes at the atomic level upon agonist and antagonist binding to GPCRs (McRobb et al., 2016; Miao and McCammon, 2016; Latorraca et al., 2017). The enhanced sampling method, metadynamics (Laio and Parrinello, 2002; Micheletti et al., 2004), supplies an effective and reliable way to explore the binding and unbinding of ligand from GPCRs, and conformational dynamics of GPCRs binding different ligands (Li et al., 2013; Schneider et al., 2015; Saleh et al., 2017). Bai et al performed metadynamics simulation on Smo-vismodegib complex to explore the binding mechanism between the vismodegib and Smo (Bai et al., 2014).

Herein we performed metadynamics simulation to shed light on the mechanism that vismodegib allosterically inhibits the activation of human Smo and binding of cholesterol, by analyzing the synergistic interaction of multiple domains of Smo. Our results revealed that the movements of TM5, TM6, and TM7 induced by vismodegib binding are crucial in deactivation of Smo. In the extracellular side, upon vismodegib binding, the hydrophobic pocket accommodating cholesterol, forming by hydrophobic residues of CRD groove, TM6, ECL3, and HD, rearrange results from the movement of TM6. Hence, the CRD groove takes the open conformational state in the inactivation state, accommodating the TM6 and ECL3. In the intracellular side of inactivation of Smo by vismodegib binding, the movements of TM3, TM5, TM6, and TM7 lead the ICL2 showing closed, ICL3 being open, and the ICL1 and W535 being away from each other. And the HD and ECLs play crucial roles in coordinating the synergistic interaction between the multiple domains of Smo in deactivation. The revealed deactivation mechanism of Smo and conformational changes will be helpful to develop of more effective modulators of Smo or detect potential active site.

## Materials and Methods

### Preparation of Simulation Systems

As a starting point in the simulations, and as a reference conformation to analyze the results, we used previously determined structures of the human Smo bound to vismodegib and cholesterol (Byrne et al., 2016). The crystal structures were obtained from the PDB database (PDB ID: 5L7I, 5L7D). The chain A of the Smo-cholesterol (hereafter called Smo-CLR) complex was selected, while the chain B of Smo-vismodegib (hereafter called Smo-Vismod) complex was selected. The cytochrome B-562 and solvate molecules of the crystal construct were omitted except water molecules. The missing residues of Smo-Vismod and Smo-CLR were built in the Protein Preparation Wizard in Schrodinger 2015 (Madhavi Sastry et al., 2013). Side chain ionization states were modeled with the PROPKA tool (Søndergaard et al., 2011). The membrane around the transmembrane domain of Smo was built by 85 × 85 Å POPC: cholesterols with 9:1 using CHARMM-GUI webserver (Lee et al., 2016), the receptor crystal structure pre-aligned in the OPM (Orientations of Proteins in Membranes) database (Lomize et al., 2006). Each system was solvated by 12 Å with a truncated rectangular box of TIP3P waters (Jorgensen et al., 1983) and neutralized to a concentration of 0.15 M NaCl.

The proteins were modeled using the AMBER FF99SB force field (Hornak et al., 2006), the ligands were modeled using the generalized AMBER force field (GAFF) (Wang et al., 2004). Geometry optimization and the electrostatic potential calculations on the ligands were performed at the HF/6-31G^*^ level in the Gaussian09 software (Frisch et al., 2009), and the partial charges were calculated with the RESP (Fox and Kollman, 1998). The force field parameters for the ligands were created by the Antechamber package.

Before metadynamics simulation, the energy minimization and equilibration were conducted by NAMD 2.9 simulation package (Phillips et al., 2005) in order to equilibrate the systems. Firstly, to remove bad contacts in the initial structures, steepest descent was carried out. After energy minimization, each system was gradually heated in NVT ensemble from 0 to 300 K in 300 ps. Subsequently, constant temperature equilibration at 300 K for a total of 5 ns was performed to adjusting the solvent density. Finally, 20 ns conventional molecular dynamic simulations were carried out for each system in NPT ensemble with periodic boundary conditions; an integration step of 2 fs was used. The particle mesh Ewald (PME) algorithm (Darden et al., 1993) was employed to treat long-range electrostatic interactions, while the non-bonded interactions were calculated based on a cutoff of 12 Å. The SHAKE algorithm (Ryckaert et al., 1977) was applied to constrain all covalent bonds involving hydrogen atoms.

### Metadynamics Simulations

Metadynamics is an efficient enhanced sampling method, allows the system to escape from local minima in the free energy surface (FES) to explore the conformational space by filling the minima with an external history-dependent bias potential, and permits an accurate determination of the FES (Laio and Parrinello, 2002; Laio and Gervasio, 2008). This bias potential is built as a sum of Gaussians deposited along the trajectory in the pre-defined collective coordinates (CVs) space.

(1) $$\begin{array}{r} V\left(\vec{s},t\right)=\underset{k\tau <t}{\sum }W\left(k\tau \right)exp\left(-\overset{d}{\underset{i=1}{\sum }}\frac{{\left(s_{i}-s_{i}\left(q\left(k\tau \right)\right)\right)}^{2}}{2{\sigma_{i}}^{2}}\right) \end{array}$$

where $V\left(\vec{s},t\right)$ is the bias potential added to the system, τ is the Gaussian deposition stride, σ_*i*_ is the width of the Gaussian for the *ith* CV, and *W*(*kτ*) is the height of the Gaussian. The effect of the metadynamics biased potential is to push the system away from local minima into visiting new regions of the phase space. Furthermore, in the long time limit, the bias potential converges to minus the free energy as the function of the CVs:

(2) $$\begin{array}{r} V\left(\vec{s},t\to \infty \right)=-F\left(\vec{s}\right)+C \end{array}$$

In standard metadynamics, Gaussians of constant height is added for the entire course of a simulation. As a result, the system is eventually pushed to explore high free-energy regions and the estimate of the free energy calculated from the bias potential oscillates around the real value.

In this work, we carried out 100 ns metadynamics simulations for each system, biasing the potential along the following two CVs: the distance between the center mass of W109 and R161, as well as the distance between the center mass of P263 and W535, ignoring hydrogen atoms and labeled as d1, d2, respectively. A Gaussian width of 0.15 Å was used for both CVs, and a Gaussian deposition rate of 0.1 *kcalmolps* was used.

## Results and Discussions

### Determination of Conformational States From Free Energy Surface

**Figure 1:**
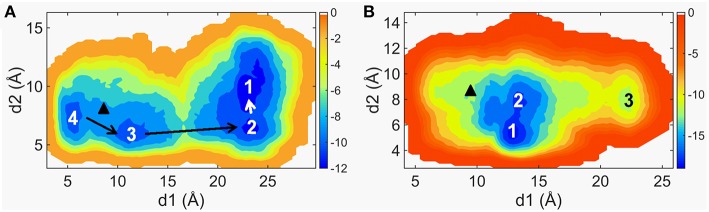
The two-dimensional maps of the free energy surfaces along d1 and d2 of Smo-Vismod **(A)** and Smo-CLR **(B)**. The marked black triangles represent the position of d1 and d2 of the crystal structures.

W109 and R161 of CRD groove located in the opposite place are used to characterize the conformational dynamics of the groove site, and they are involved in binding of sterols (Rana et al., 2013). P263 located in the C-terminal of ICL1 and W535 located in the intercellular tip of TM7. The communication between ICL1 and W535 may trigger Smo activation (Arensdorf et al., 2016), since the activating Smo mutation W535L has been already known in BCCs (Xie et al., 1998; Lam et al., 1999). The free energy surfaces (FES) are shown in Figure 1. The FES along d1 and d2 achieves convergence in last 10 ns for each system (Supplementary Figure 1). As seen in Figure 1A, the FES of Smo-Vismod in the spatial distribution is separated obviously by four energy basins, marked as *1, 2, 3*, and *4*. While the FES of Smo-CLR owns two energy basins and a minor energy basin, marked as *1, 2*, and *3* (Figure 1B). That is, the Smo-Vismod undergoes larger conformational changes compared with Smo-CLR. As a comparison, we marked the location of crystal structures in the FES diagram according to the values of d1 and d2 (Figure 1). Obviously, the crystal structures of Smo-Vismod and Smo-CLR sit among the intermediates and finally stabilize, respectively, in state *1* with the simulations.

Furthermore, d1 varies widely of Smo-Vismod while d2 varies in a narrow range both in Smo-Vismod and Smo-CLR. We can still differentiate the transformation among states (Figure 1). By combining the FES distribution of Smo-Vismod and Smo-CLR (Figure 1), as well as the crystal structures of Smo bound to different agonist or antagonist (Nachtergaele et al., 2013; Rana et al., 2013; Wang et al., 2014; Weierstall et al., 2014; Byrne et al., 2016; Huang et al., 2016; Zhang et al., 2017) (Supplementary Table 1), we speculated that the CRD groove of the inhibited state is open. ICL1 and TM7 are away from each other, with the corresponding d1 is 23 ± 3 Å and d2 is 10 ± 2 Å. The d1 and d2 shifting to 12 ± 2 Å and 5 ± 1 Å are considered as activated state of Smo, the CRD groove is in a closed conformation where can accommodate cholesterol, and ICL1 and TM7 remain communicating. The d1 varying between 17 ± 3 or 7 ± 2 Å indicates the intermediate states of Smo, meanwhile, the d2 varies between 7 ± 1 Å (Table 1).

**Table 1:** The conformational states of Smo were determined by d1 and d2.

**State**	**d1 (Å)**	**d2 (Å)**
Inhibition	23 ± 3	10 ± 2
Activation	12 ± 2	5 ± 1
Intermediate	17 ± 3/7 ± 2	7 ± 1

### The Conformational Difference Between Smo-Vismod and Smo-CLR

**Figure 2:**
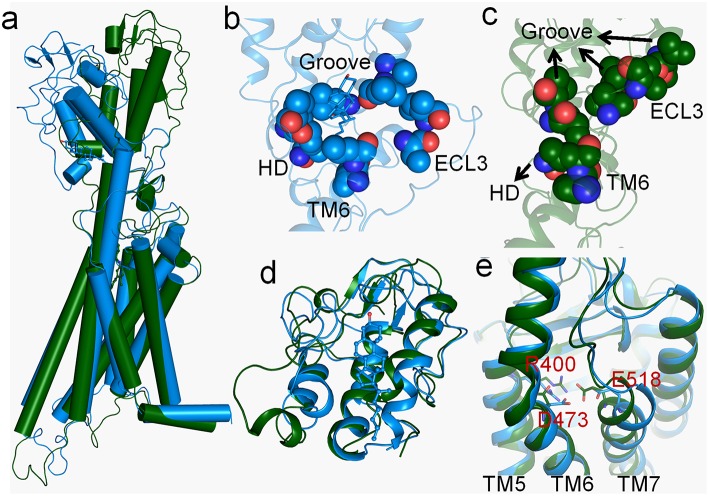
**(a)** The representative structures of Smo-Vismod (forest) and Smo-CLR (marine) at the free-energy minima. **(b,c)** Hydrophobic interaction formed among CRD groove, HD, TM6 and ECL3 of Smo-CLR and Smo-Vismod respectively; **(d)** the conformational comparison of CRDs between Smo-Vismod and Smo-CLR; **(e)** conformational comparison of TMD-sites between Smo-Vismod and Smo-CLR.

As marked in Figure 1, Smo-Vismod and Smo-CLR transform from similar conformation to their respective stable conformations. In order to observe the conformational difference of stable states between Smo-Vismod and Smo-CLR, we extracted the representative structures from the global minimum of FES, respectively. As shown in Figure 2a, the most significant difference is that the CRD tilts to the membrane plane of Smo-CLR, almost forms 60° angle between CRD and the membrane plane. Contrarily, the CRD of Smo-Vismod further moves away from the membrane plane and almost perpendicular to the membrane plane. The distance of the center mass between CRDs of Smo-Vismod and Smo-CLR is 15.6 Å by aligning their TMDs. Compared with their crystal structures (Supplementary Figure 2), the CRD of Smo-CLR slightly tilts to membrane plane related to Smo-Vismod with respect to the aligned TMDs, and the distance of the center mass between CRDs of Smo-Vismod and Smo-CLR is 6.0 Å. This means that their crystal structures are not the stable states, both of the structures undergo substantial conformational rearrangement.

To investigate the details of conformational difference between the Smo-Vismod and Smo-CLR, we compared the CRD groove sites and TMD sites of the Smo-Vismod and Smo-CLR (Figures 2b–e). Firstly, we studied the cholesterol binding site, a hydrophobic pocket formed by the hydrophobic residues in CRD (residues V107, L108, L112, I156, V157), HD (residue V210), TM6 (residues V488, L489) and ECL3 (residues V494, I496) (Supplementary Figure S2B). And the hydrophobic pocket is still maintained by cholesterol binding in Smo-CLR (Figure 2b). However, in the stable state of Smo-Vismod, the extracellular extension of TM6 and ECL3 occupy the CRD groove (Figure 2c) and the CRD groove site collapses. The CRD groove, HD, TM6 and ECL3 form strong hydrophobic interactions. Therefore, TM6 and ECL3 occupy the CRD groove in Smo-Vismod, which hinders the cholesterol binding to groove site.

The aligned CRDs of stable states of Smo-Vismod and Smo-CLR (Figure 2d), the CRD groove of Smo-Vismod is open, while the CRD groove of Smo-CLR is closed relatively. However, in the crystal structures, the conformations of CRDs of Smo-Vismod and Smo-CLR are similar (Supplementary Figure S2C), the RMSD is about 0.30 Å, in the closed state. The open conformation of CRD groove induced by the vismodegib bound in our simulation, except the CRD-apo structure of *Xenopus laevis* Smo has a similar open conformation (Supplementary Table 1) (Huang et al., 2016), exists neither in other CRD crystal structures nor in the multiple-domain crystal structures that resolved previously. Obviously, the CRD groove of inactivated Smo takes the open conformation, which accommodates TM6 and ECL3 by forming strong hydrophobic interactions. In the TMD-site (Figure 2e), the notable variations are the movements of TM5, TM6, and TM7. Compared with Smo-CLR, the TM6 of Smo-Vismod moves outward from the helix bundle, while TM5 and TM7 move inward (Figure 2e).

### The Conformational Dynamics of the CRD Groove Site

**Figure 3:**
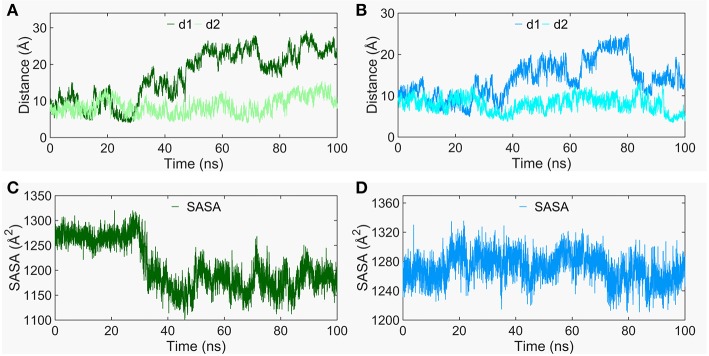
The evolution of d1 and d2 along time of Smo-Vismod **(A)** and Smo-CLR **(B)**. The solvent accessible surface area (SASA) of the hydrophobic pockets consisted of hydrophobic residues of CRD groove, HD, helix VI and ELC3 evolve with time for Smo-Vismod **(C)** and Smo-CLR **(D)**.

The Smo-Vismod undergoes significant conformational changes during the simulation (Figures 1, 2). We analyzed the conformational dynamics process of Smo-Vismod from the FES (Figure 1). Based on the hydrophobicity of the CRD groove site, and the interaction among the hydrophobic residues of CRD groove, HD, TM6, and ECL3, the solvent-accessible surface (SASA) of the hydrophobic pocket composed of them is observed over time (Figures 3C,D), along with the evolution of d1 (Figures 3A,**B**) to study the conformational dynamics of the ECD of Smo-Vismod.

**Figure 4:**
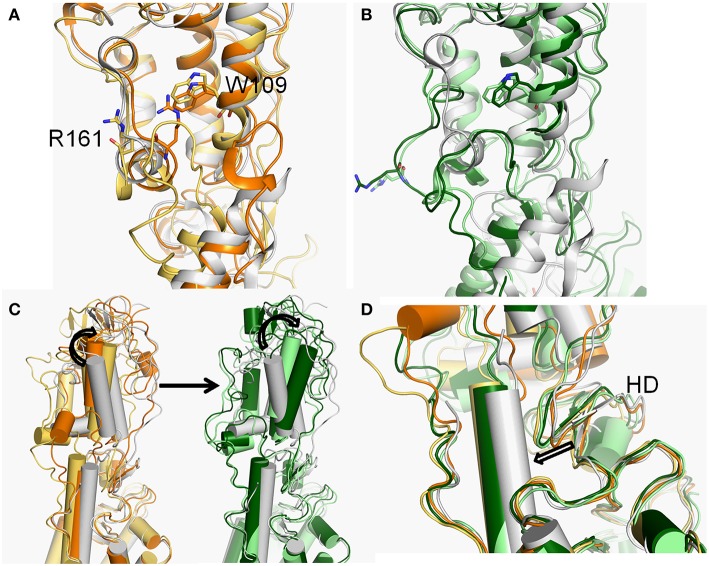
**(A,B)** The conformational dynamics of ECDs of basins *4* (orange), *3* (yellow-orange), *2* (light green), and *1* (forest) of Smo-Vismod compared with the crystal structure (white). **(C)** The conformational changes of the CRD. **(D)** The conformational changes of HD.

At the ~30 ns of the start of the simulation, the d1 is <15 Å (Figure 3A), and the SASA of the hydrophobic pocket during the simulation is not significant change (Figure 3C), indicating that the CRD groove is still in the closed state (Supplementary Table 1). The corresponding energy basin is *4* (Figure 1A). The representative conformation of the energy basin *4* was superimposed with the crystal structure of the Smo-Vismod (Figure 4A). As seen that the CRD groove is in a closed state as in the initial state. The extracellular extension of TM6 and ECL3 do not occupy the CRD groove, nor form hydrophobic interaction. Compared to the initial structure, TM6 and ECL3 have begun to approach the groove.

The second obvious fluctuation is from ~30 to ~50 ns, the d1 increases rapidly and fluctuates in the range of ~15 to ~20 Å. The corresponding SASA of the hydrophobic pocket decreases rapidly, indicating that the CRD groove is beginning to open. The appropriate energy basin is *3* (Figure 1A). As shown in Figure 4A, we found that TM6 and ECL3 enter into the CRD groove. The hydrophobic pockets formed among the hydrophobic residues of CRD, HD, TM6, and ECL3 begin to be closed. Several hydrophilic residues (E158-G162) of the side of CRD groove begin to leave the groove site and hence the CRD groove gets gradually open.

At the end of 50 ns, d1 increases and fluctuates between 20 and 25 Å during the subsequent simulation (Figure 3A). The SASA of the hydrophobic pocket also fluctuates at a steady level in the last 50 ns (Figure 3C), indicating that the hydrophobic pocket maintains in compact contact. The representation of energy basins are *1* and *2* (Figure 1A). Compared the representative structures of the two energy basins (Figure 4B), the TM6 and ECL3 have completely occupied the CRD groove and form strong hydrophobic interactions with the CRD groove. CRD groove presents a significant open conformation. With the TM6 and ECL3 of Smo-Vismod gradually approach to CRD groove, CRD is away from the membrane plane (Figure 4C), the HD moves toward the direction of TM6 (Figure 4D). The synergistic movement of the extracellular domain of Smo-Vismod contributes to stabilize CRD in inactivated conformation.

In contrast, the d1 smoothly fluctuates at the beginning of ~25 ns; after ~25ns, mainly fluctuates between 10 and 15 Å (Figure 3B); the SASA shows increased at beginning ~ 20 ns (Figure 3D) in Smo-CLR. We compared the representative structures of Smo-CLR in the energy basins (Supplementary Figure S3). It is clear that CRD tilts toward the membrane plane during the simulations, the TM6 shifts toward the flank of CRD, simultaneously, the ECL3 deviates from CRD. And the HD moves away from TM6 (Supplementary Figure S3A). However, the CRD groove does not change significantly (Figure S3B). Noticeably, the activated conformational dynamics of ECD is in contrast to the inhibited conformation of Smo-Vismod.

At this point, we have proved that the movement of TM6 and the hydrophobic residues of CRD groove, HD, TM6, and ECL3 play crucial roles in the deactivation of Smo by vismodegib binding. Indeed, the TM6 and ECL3 occupy the CRD groove in Smo-Vismod, which hinders the cholesterol binding to groove site.

### The Conformational Dynamics of the TMD-Site

**Figure 5:**
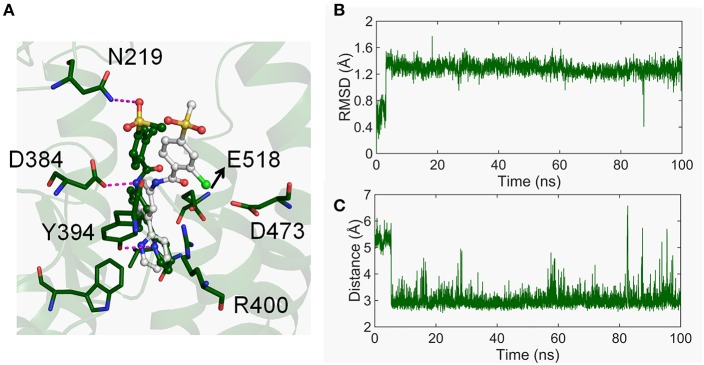
**(A)** The binding mode of vismodegib of the represent structure in free energy minima (forest) varied compared with the crystal structure (white). **(B)** The RMSD of heavy atoms of vismodegib evolved along with time. **(C)** The distance between the atom O3 of vismodegib and the atom ND2 of N219 evolved along with time.

As observed above, the movement of TM6 is crucial in the conformational dynamics of the CRD groove site. Considering the binding mode of vismodegib that directly interacts with TM5, TM6, and TM7, we firstly studied the interaction between vismodegib and TMD-site. As shown in Figure 5A, we observed that the chlorophenyl–methylsulfone moiety of vismodegib overturns nearly 90° in the stable inactivated state and closes to HD-ECL2 compared with the crystal structure. The atom O3 of the methylsulfone moiety forms H-bond with atom ND2 of side-chain of N219. The RMSD of vismodegib was calculated throughout the simulation (Figure 5B). We caught sight of the obvious remolding, that is, the RMSD suddenly increases from ~0.4 to ~1.4 Å at the beginning ~5 ns, and fluctuates stably between 1.2 and 1.6 Å, indicating that the binding mode of vismodegib remodels immediately during the simulation. We also monitored the distance between atom O3 of the methylsulfone moiety and atom ND2 of N219 (Figure 5C). The distance rapidly drops to ~3 Å from around 5.2 Å at the beginning simulation of ~5 ns and eventually runs aground. This means that the vismodegib rapidly leaves the initial binding mode shortly after the start of the simulation, afterwards, stabilizes in the new binding mode, forming steady H-bond with side-chain of N219, which also indicates the importance of HD.

**Figure 6:**
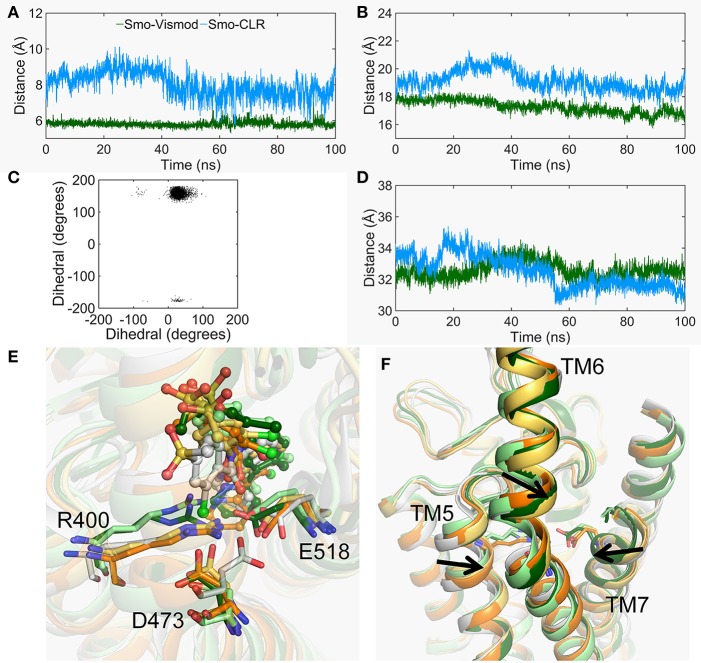
The conformational changes of TMD-site. **(A)** The center mass distance between the side chain of R400 and E518 evolved along with time in the Smo-Vismod (forest) and Smo-CLR (marine). **(B)** The distance of the extracellular end of TM5 (residues 397-401) and TM7 (residues 515-519) evolved along with time. **(C)** The scatter plot of dihedral of C, CA, CB, and CG of D473 between the Smo-Vismod and Smo-CLR. **(D)** The distance of the extracellular end of TM3 (residues 315-319) and TM6 (residues 488-492) evolved along with time. **(E)** The conformational changes of R400, D473 and E518. **(F)** The movements of TM5, TM6, and TM7.

At the other side of TMD-site, the charged residue R400 of TM5, D473 of TM6, and E518 of TM7 contribute to the binding of vismodegib (Wang et al., 2014; Byrne et al., 2016). The movement of TM5, TM6, and TM7 is obvious in the stable states of Smo-Vismod and Smo-CLR (Figure 2e). Therefore, we analyzed the conformational dynamics of the three polar residues (Figure 6). As seen in Figure 6A, the distance between R400 and E518 of Smo-Vismod is much smaller than Smo-CLR throughout the simulation. This indicates that the two residues going close to each other at the beginning of the simulation and form stable electrostatic interaction Smo-Vismod, which results in TM5 and TM7 moving inward (Figures 6B,F). TM6 is extruded from the helix bundle (Figure 6F). At the same time, the two residues going close to each other results in a remolding binding mode of vismodegib. Since in the crystal structure, the negatively charged E518 is near the amide oxygen atom of vismodegib with the distance of 3.1 Å from OE2 of E518 (Supplementary Figure S4), the electrostatic repulsion causes overturning of vismodegib.

In addition, due to R400 and E518 going close to each other, the electrostatic repulsion of the E518 and the electrostatic attraction of the R400 result in the D473 deflected (Figures 6C,E). The dihedral angles of the atoms C, CA, CB, and CG of the D473 of the Smo-Vismod is far less than the Smo-CLR (Figure 6C). D473 points toward R400 in the representative state of Smo-Vismod (Figure 6E) instead of pointing toward E518 in the crystal structure (Supplementary Figure S4).

From these results, the remodeled binding model of vismodegib is stabilized by forming H-bonds with N219 of HD, D384, and Y394 of ECL2, respectively. Additionally, R400, E518, and D473 constitute the electrostatic interface to form polar interaction with amide linker of vismodegib (Figures 5A, 6E). R400, D473, and E518 play a vital role in the conformational dynamics of helices. Moreover, the outward movement of TM6 makes the extracellular extension of TM6 close to the CRD groove, and thus along with ECL3 interact with hydrophobic residues of CRD groove and HD, which hinders the binding of the cholesterol. On the contrary, cholesterol occupied the groove to push the ECL3 away from the groove and keep the TM6 out of the groove, allowing the CRD to tilt toward membrane plane.

### The Conformational Dynamics of the ICD

**Figure 7:**
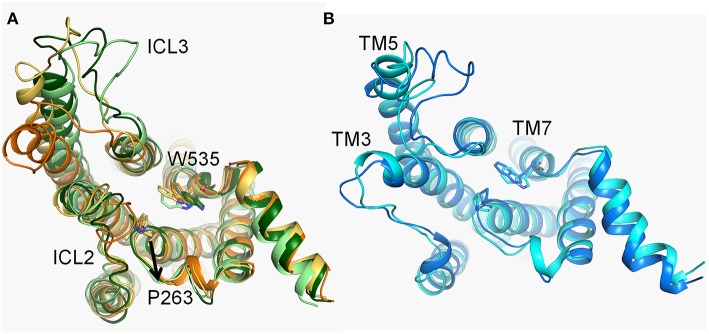
**(A)** The conformational dynamics of ICDs of basins *4* (orange), *3* (yellow-orange), *2* (light green), and *1* (forest) in the Smo-Vismod. **(B)** The conformational dynamics of ICDs of basins *1* (marine) and *2* (cyan) in Smo-CLR.

Compared with d1, the fluctuation of d2 is not significant throughout the simulation in both of systems (Figures 3A,B). Nevertheless, three major energy basins of Smo-Vismod along d2, termed to *4, 2*-*3* and *1*, were obtained (Figure 1A). We compared the representative structures of the intracellular domain of Smo-Vismod and Smo-CLR, the ICLs give notable variations (Figure 7). Compared with Smo-CLR, the intracellular end of TM7 of Smo-Vismod shifts outward. Therefore, there is no communication between ICL1 and W535, which indicates an inhibited state of Smo. Furthermore, the significant conformational changes are the ICLs. As shown in Figure 7A, the intracellular end of TM3 shifts inward and ICL2 covers over the central interface of the helix bundle showing closed in the four representative structures of Smo-Vismod. The ICL3 is opening with the simulation due to the shifts of intracellular tips of TM5 and TM6. While, the ICL2 is open, and the ICL3 is closed in the Smo-CLR (Figure 7B). The importance of ICLs of Smo in regulating Smo signaling was confirmed by peptide mimics of ICL2 and ICL3 that suppress Smo induced tumor cell proliferation (Remsberg et al., 2007). In our simulations, the closed ICL2 and the open ICL3 are only seen in the Smo-Vismod, which proves that the intracellular loops potentially affect the interaction of downstream effectors and Smo. Compared the representative conformations of Smo-Vismod and Smo-CLR, we have got an outline of inhibited Smo induced by vismodegib. The vismodegib binding to TMD site leads to the movement of TM6, which promotes the extracellular extension of TM6 and ECL3 to occupy the groove by forming strong hydrophobic interactions (Figure 2c). Hence the vismodegib binding blocks the binding of cholesterol. At the intracellular side, the ICL2 shows closed owing to the shifts of TM3. ICL3 is open caused by the shifts of TM5 and TM6. The ICL1 and W535 are away from each other due to the TM7 shifts outward. The interaction of the multi-domain of Smo induced by vismodegib hinders the binding of the cholesterol, and destroys the intracellular interactional interface of the effector.

### The Communication of Multi-Domain of Smo

**Figure 8:**
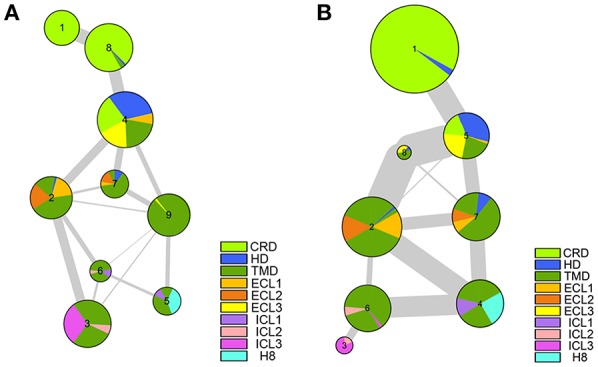
The communication between the multiple domains of Smo-Vismod **(A)** and Smo-CLR **(B)**.

We have made it clear so far that the antagonist vismodegib binding leads to the movements of TM5, TM6, and TM7, and then stabilizes Smo in an inactive state through coordinated movement between multiple domains. We further carried out the dynamical network analysis (Sethi et al., 2009) to investigate the communication of multi-domain of Smo in the inactive state and active state. As shown in Figure 8, the HD, ECLs spread in multiple communities in both of the systems, indicating these domains play a key role in coordinating the interaction of CRD and TMD. Which owe to the inherent flexibility and naturally ingenious arrangement of these domains. And the Smo-Vismod identifies more communities, and the communities more weakly connect each other compared with Smo-CLR, suggesting that the construction of Smo is looser induced by vismodegib. While the Smo-CLR takes a compact and ordered construction ensuring the extracellular signal transmits to the intracellular side and permitting the downstream effectors to contact.

We have demonstrated the vismodegib binding triggers the shifts of TM5, TM6, and TM7, leading the extracellular extension of TM6 and ECL3 to enter the CRD groove and block the binding of cholesterol. Simultaneously, the significant conformational changes of ICLs are attributed to the movements of TM3, TM5, TM6, and TM7. The communication of multi-domain of Smo proves that HD and ECLs play roles in the synergistic movements of CRD and TMD. The looser construction and weak communication provide the view of the inactive state of Smo.

## Conclusions

In this work, the mechanism of the vismodegib allosterically inhibits Smo activation and hinders cholesterol binding is revealed by metadynamics simulations. We revealed that the vismodegib binding leads movements of TM5, TM6, and TM7, and the shift of TM6 triggers the entrance extracellular extension of TM6 and ECL3. Therefore, the TM5, TM6, and TM7 are key factors to the deactivation of Smo upon vismodegib binding. Moreover, we also found an inhibited open conformation of CRD groove, which is not shown in crystal structures. The open CRD groove accommodates the TM6 and ECL3 so that hinders cholesterol to bind and holds the CRD stacked atop the TMD. The strong hydrophobic interaction of CRD groove, HD, TM6 and ECL3 stabilizes the interaction of them. Furthermore, the HD and ECLs play a key role in the coordinated interaction of CRD, TMD and themselves. Therefore, blocking the coordinated movement of CRD, HD, and ECLs may potentially inhibit the activation of Smo. And we observed a remolding of vismodegib binding, stabilized by hydrogen bond formed with N219, D384 and Y394. These results can be taken into account for the design and discovery of novel Smo inhibitors, or providing structural information for discovering potential active sites.

## Author Contributions

XA, QB, FB, and DS designed the research, performed the dynamic simulations and analyzed the data. XA and XY wrote the manuscript. HL and XY directed the project.

## Conflict of Interest Statement

The authors declare that the research was conducted in the absence of any commercial or financial relationships that could be construed as a potential conflict of interest.
