# Supplementary material for: Deciphering the Allosteric Effect of Antagonist Vismodegib on Smoothened Receptor Deactivation Using Metadynamics Simulation
Source: Front Chem. 2019 Jun 4;7:406. doi: 10.3389/fchem.2019.00406 (PMC6558189; doi:10.3389/fchem.2019.00406)
Supplement: Supplementary file 1 [file Table_1.DOCX]

Supplementary Material

# Supplementary Figures and Tables

**1.1 Supplementary Tables**

**Table. S1** The d1 and d2 of crystal structures of Smo in CRD or multi-domains

| PDB code | d1 (Å) | d2 (Å) | state |
| --- | --- | --- | --- |
| 4c79 | 7.3 |  | Z_Smo-apo |
| 4c7a | 7.8 |  | Z_Smo-apo (selenomethionine) |
| 5kzv | 10.2 |  | X_Smo-agonist |
| 5kzz | 16.3 |  | X_Smo-apo |
| 4jkv |  | 8.9(A)/8.6 (B) | H_Smo-antagonist |
| 4n4w |  | 9.0 | H_Smo-antagonist |
| 4o9r |  | 8.8 | H_Smo-antagonist |
| 4qim |  | 9.4 | H_Smo-antagonist |
| 4qin |  | 7.2 | H_Smo-agonist |
| 5l7d | 9.5 (A)/10.5 (B) | 8.5 (A)/8.5 (B) | H_Smo-agonist (A)/H_Smo-apo (B) |
| 5l7i | 7.9 (A)/8.6 (B) | 7.9 (A)/7.9 (B) | H_Smo-antagonist |
| 5v56 | 8.7 (A)/7.5 (B) | 9.2 (A)/9.7 (B) | H_Smo-antagonist |

**1.2 Supplementary Figures**


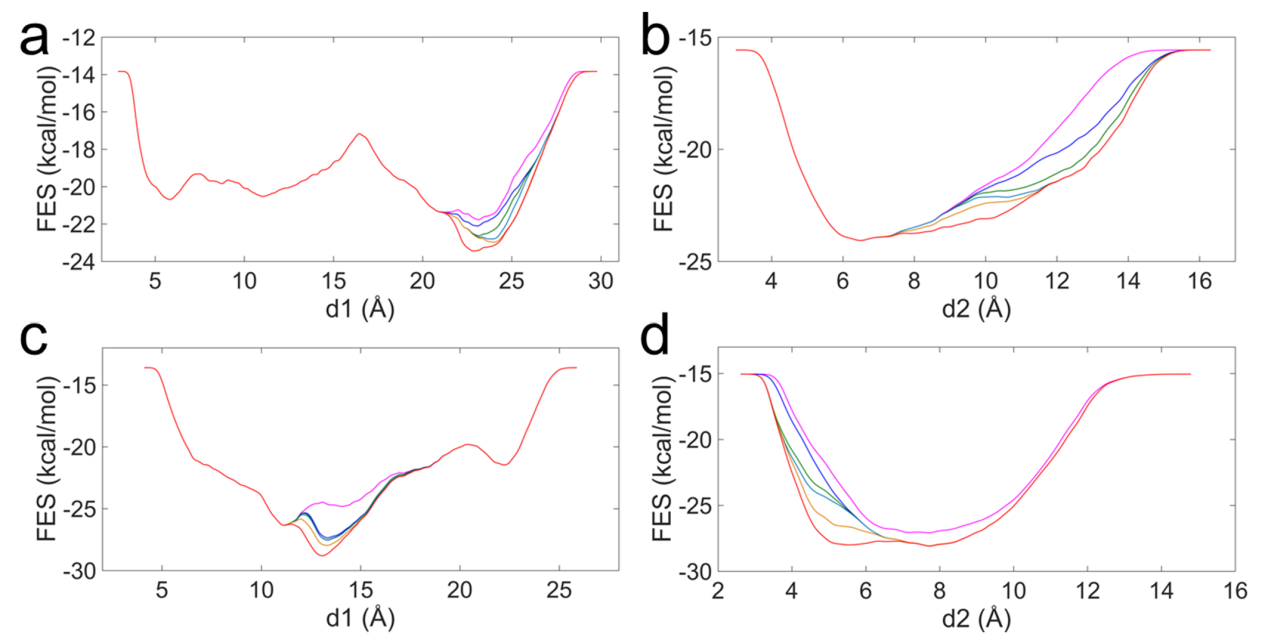


**Supplementary Figure 1.** The free energy distribution along d1 and d2 respectively at 90ns (magenta), 95 ns (blue), 97 ns (green), 98 ns (marine), 99 ns (orange) and 100 ns (red) of Smo-Vismod (a b) and Smo-CLR (c d).


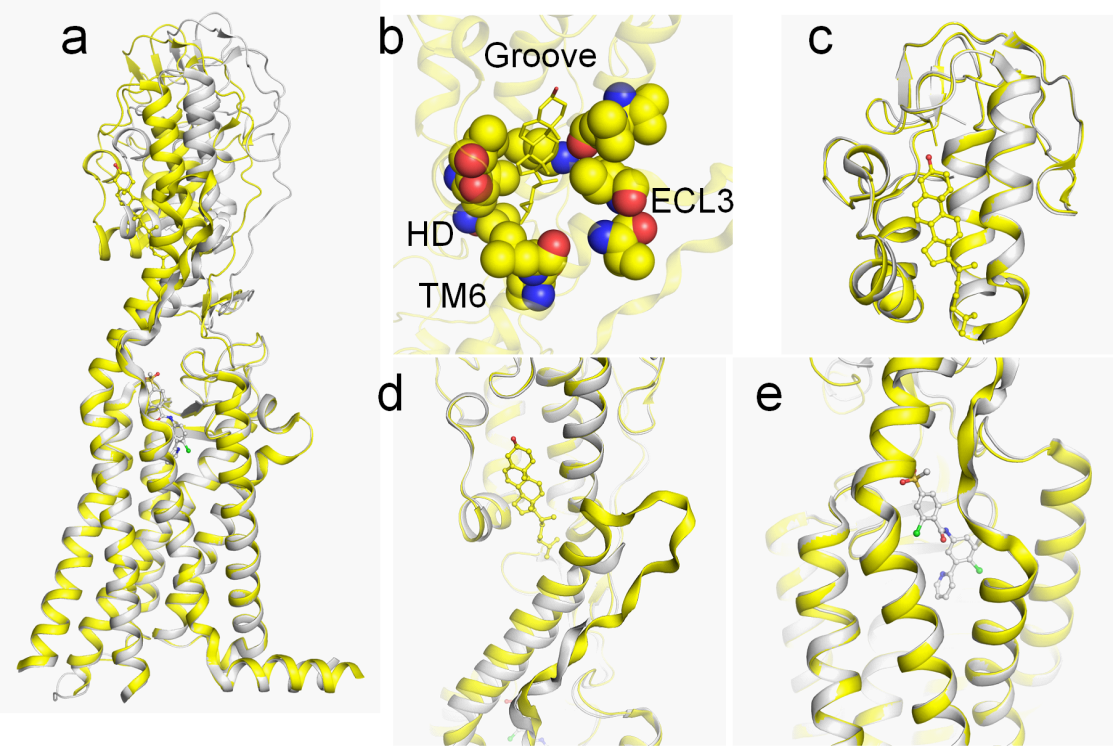


**Supplementary Figure 2.** (a) Aligning the crystal structures of Smo-Vismod (white) and Smo-CLR (yellow). (b) The hydrophobic pockets consisted of CRD groove, HD, TM6 and ECL3. (c, d, e) The conformations of CRD grooves, TM6 and ELC3, TMD-site of Smo-Vismod and Smo-CLR respectively.


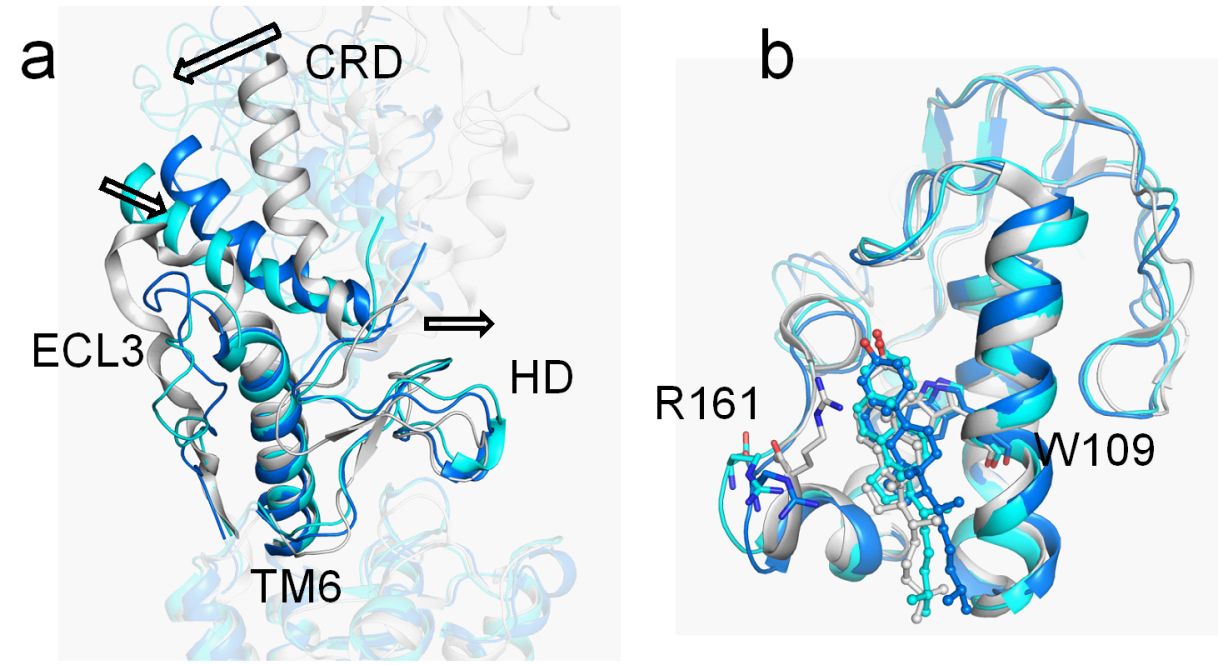


**Supplementary Figure 3.** The representative conformation of basins 1 (marine) and 2 (cyan) of Smo-CLR compared with crystal structure (white). (a) The conformational dynamics of CRD, TM6, ECL3 and HD; (b) The conformational dynamics of CRD groove.


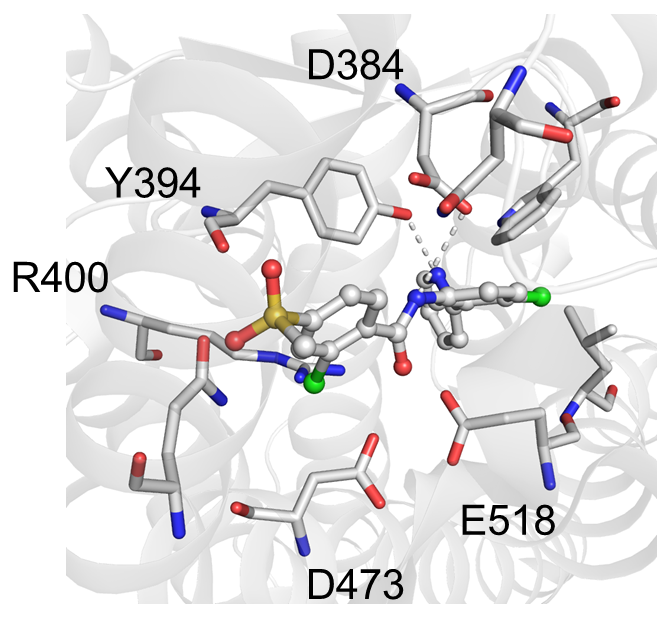


**Supplementary Figure 4.** The binding mode of vismodegib in crystal structure.
